# Association Between Residential Greenness, Cardiometabolic Disorders, and Cardiovascular Disease Among Adults in China

**DOI:** 10.1001/jamanetworkopen.2020.17507

**Published:** 2020-09-21

**Authors:** Bo-Yi Yang, Li-Wen Hu, Bin Jalaludin, Luke D. Knibbs, Iana Markevych, Joachim Heinrich, Michael S. Bloom, Lidia Morawska, Shao Lin, Pasi Jalava, Marjut Roponen, Meng Gao, Duo-Hong Chen, Yang Zhou, Hong-Yao Yu, Ru-Qing Liu, Xiao-Wen Zeng, Mohammed Zeeshan, Yuming Guo, Yunjiang Yu, Guang-Hui Dong

**Affiliations:** 1 Guangdong Provincial Engineering Technology Research Center of Environmental and Health Risk Assessment, Department of Occupational and Environmental Health, Sun Yat-sen University School of Public Health, Guangzhou, China; 2 Centre for Air Quality and Health Research and Evaluation, Glebe, New South Wales, Australia; 3 Population Health, South Western Sydney Local Health District, Liverpool, New South Wales, Australia; 4 Ingham Institute for Applied Medical Research, Liverpool, New South Wales, Australia; 5 University of New South Wales School of Public Health and Community Medicine, Kensington, New South Wales, Australia; 6 University of Queensland School of Public Health, Herston, Queensland, Australia; 7 Institute and Clinic for Occupational, Social and Environmental Medicine, University Hospital, LMU Munich, Munich, Germany; 8 Institute of Epidemiology, Helmholtz Zentrum Munchen–German Research Center for Environmental Health, Neuherberg, Germany; 9 Comprehensive Pneumology Center Munich, German Center for Lung Research, Munich, Germany; 10 Department of Environmental Health Sciences, University at Albany, State University of New York, Rensselaer; 11 Department of Epidemiology and Biostatics, University at Albany, State University of New York, Rensselaer; 12 International Laboratory for Air Quality and Health, Queensland University of Technology, Brisbane, Queensland, Australia; 13 Department of Environmental and Biological Sciences, University of Eastern Finland, Kuopio, Finland; 14 Department of Geography, Hong Kong Baptist University, Hong Kong SAR, China; 15 Guangdong Environmental Monitoring Center, State Environmental Protection Key Laboratory of Regional Air Quality Monitoring, Guangdong Environmental Protection Key Laboratory of Atmospheric Secondary Pollution, Guangzhou, China; 16 Department of Epidemiology and Preventive Medicine, Monash University School of Public Health and Preventive Medicine, Melbourne, Victoria, Australia; 17 State Environmental Protection Key Laboratory of Environmental Pollution Health Risk Assessment, South China Institute of Environmental Sciences, Ministry of Environmental Protection, Guangzhou, China

## Abstract

**Importance:**

Living in areas with more vegetation (referred to as residential greenness) may be associated with cardiovascular disease (CVD), but little data are available from low- and middle-income countries. In addition, it remains unclear whether the presence of cardiometabolic disorders modifies or mediates the association between residential greenness and CVD.

**Objective:**

To evaluate the associations between residential greenness, cardiometabolic disorders, and CVD prevalence among adults in China.

**Design, Setting, and Participants:**

This analysis was performed as part of the 33 Communities Chinese Health Study, a large population-based cross-sectional study that was conducted in 33 communities (ranging from 0.25-0.64 km^2^) in 3 cities within the Liaoning province of northeastern China between April 1 and December 31, 2009. Participants included adults aged 18 to 74 years who had resided in the study area for 5 years or more. Greenness levels surrounding each participant’s residential community were assessed using the normalized difference vegetation index and the soil-adjusted vegetation index from 2010. Lifetime CVD status (including myocardial infarction, heart failure, coronary heart disease, cerebral thrombosis, cerebral hemorrhage, cerebral embolism, and subarachnoid hemorrhage) was defined as a self-report of a physician diagnosis of CVD at the time of the survey. Cardiometabolic disorders, including hypertension, diabetes, dyslipidemia, and overweight or obese status, were measured and defined clinically. Generalized linear mixed models were used to evaluate the association between residential greenness levels and CVD prevalence. A 3-way decomposition method was used to explore whether the presence of cardiometabolic disorders mediated or modified the association between residential greenness and CVD. Data were analyzed from October 10 to May 30, 2020.

**Main Outcomes and Measures:**

Lifetime CVD status, the presence of cardiometabolic disorders, and residential greenness level.

**Results:**

Among 24 845 participants, the mean (SD) age was 45.6 (13.3) years, and 12 661 participants (51.0%) were men. A total of 1006 participants (4.1%) reported having a diagnosis of CVD. An interquartile range (1-IQR) increase in the normalized difference vegetation index within 500 m of a community was associated with a 27% lower likelihood (odds ratio [OR], 0.73; 95% CI, 0.65-0.83; *P* < .001) of CVD prevalence, and an IQR increase in the soil-adjusted vegetation index within 500 m of a community was associated with a 26% lower likelihood (OR, 0.74; 95% CI, 0.66-0.84; *P* < .001) of CVD prevalence. The presence of cardiometabolic disorders was found to mediate the association between residential greenness and CVD, with mediation effects of 4.5% for hypertension, 4.1% for type 2 diabetes, 3.1% for overweight or obese status, 12.7% for hypercholesterolemia, 8.7% for hypertriglyceridemia, and 11.1% for high low-density lipoprotein cholesterol levels.

**Conclusions and Relevance:**

In this cross-sectional study, higher residential greenness levels were associated with lower CVD prevalence, and this association may be partially mediated by the presence of cardiometabolic disorders. Further studies, preferably longitudinal, are warranted to confirm these findings.

## Key Points

**Question:**

Is the general vegetation level of a residential area, referred to as residential greenness, associated with cardiovascular disease among adults, and does the presence of cardiometabolic disorders mediate or modify the association between residential greenness and cardiovascular disease?

**Findings:**

In this cross-sectional study of 24 845 adults in China, residential areas with higher greenness levels were associated with a lower likelihood of cardiovascular disease. The presence of cardiometabolic disorders partially mediated the association between residential greenness and cardiovascular disease.

**Meaning:**

The study’s findings may be helpful for health care professionals and policy makers in the development of strategies, such as planning for green spaces in residential areas, to mitigate the burden of cardiovascular disease.

## Teaser

This cross-sectional study uses the normalized difference vegetation index and the soil-adjusted vegetation index to examine the associations between the level of residential greenness, the presence of cardiometabolic disorders, and the prevalence of cardiovascular disease among adults in China.

## Introduction

Cardiovascular disease (CVD) remains the primary cause of death and disability worldwide, especially in low- and middle-income countries.^1^ Between 1990 and 2016, the estimated number of individuals with CVD in China increased from 40.6 million to 93.8 million.^2^ Interventions and control strategies are therefore needed to mitigate this increase in CVD prevalence.

Accumulating data indicate that a higher amount of neighborhood vegetation (referred to as residential greenness) is associated with a range of beneficial health outcomes.^3,4^ A large number of studies have examined the cardiovascular associations of greenness,^5,6,7,8,9,10,11,12,13,14,15,16,17,18,19,20,21,22,23,24,25,26,27,28,29,30,31,32,33,34,35,36,37^ but more than 50% of those focused on CVD mortality. In addition, most of the previous studies were conducted in middle- and high-income countries, and only a few studies were performed in low-income countries.^23,27,30,31^ It is unclear if the results of studies from high-income countries are generalizable to lower-income settings because a number of cultural, sociodemographic, and environmental factors differ between high-income nations vs lower-income nations.

Cardiometabolic disorders, including hypertension, type 2 diabetes, overweight or obese status, and dyslipidemia, are documented preclinical factors associated with CVD risk.^38^ In environmental epidemiologic studies, these cardiometabolic disorders have often been suggested as intervening variables in the association between environmental exposures and CVD. A systematic review and meta-analysis^3^ as well as previous findings from the 33 Communities Chinese Health Study^39,40,41^ have indicated that higher residential greenness levels were associated with a lower risk of cardiometabolic disorders. Two studies within the past 5 years have reported that the presence of cardiometabolic disorders partially mediated the association between greenness and CVD prevalence.^28,36^ However, it is also plausible that the presence of cardiometabolic disorders may modify the health outcomes associated with residential greenness because individuals with these disorders may change their lifestyles (eg, engage in physical activity or visit parks more frequently) as part of a treatment plan. Therefore, the associations between residential greenness, cardiometabolic disorders, and CVD remain unclear. To address these research gaps, we aimed to (1) examine the association between residential greenness levels and CVD prevalence in a general population of Chinese adults and (2) explore whether the presence of cardiometabolic disorders mediated or modified the association between residential greenness levels and CVD using an advanced 3-way decomposition method.

## Methods

### Participants and Procedures

The 33 Communities Chinese Health Study, a large population-based cross-sectional investigation, was conducted in the Liaoning province of northeastern China between April 1 and December 31, 2009. The province is a large industrial center that is highly urbanized. The prevalence rates of CVD and cardiometabolic disorders have been reported to be high in this region.^42,43,44^ This study followed the Strengthening the Reporting of Observational Studies in Epidemiology (STROBE) reporting guideline for cross-sectional studies. The Human Studies Committee of Sun Yat-sen University approved the study protocols. All participants provided written informed consent.

We used a 4-stage stratified cluster sampling strategy to randomly recruit study participants^39,40,41^ (Figure). First, 3 cities (Shenyang, Anshan, and Jinzhou) were randomly selected among 14 total cities in the Liaoning province. Second, 3 residential communities were randomly selected from each of the 11 total districts (5 districts in Shenyang and 3 districts each in Anshan and Jinzhou), generating 33 communities that ranged in size from 0.25 km^2^ to 0.64 km^2^. Third, 700 to 1000 households were randomly selected from each community. Fourth, 1 adult was randomly selected from each household. A total of 28 830 individuals were initially invited to participate in the study. Of those, 3985 individuals (13.8%) were excluded for the following reasons: (1) 3634 individuals did not complete the study questionnaire; (2) 233 individuals had resided in the study community for less than 5 years; (3) 79 individuals were pregnant; (4) 27 individuals were younger than 18 years or older than 74 years; and (5) 12 individuals had a serious preexisting disease, such as terminal cancer. After exclusions, 24 845 participants (86.2%) were included in the analysis, and 15 477 participants (53.7%) provided venous blood samples after fasting. Sociodemographic characteristics were similar between the participants who provided blood samples and the participants who did not (n = 9368) (eTable 2 in the Supplement).

**Figure.**
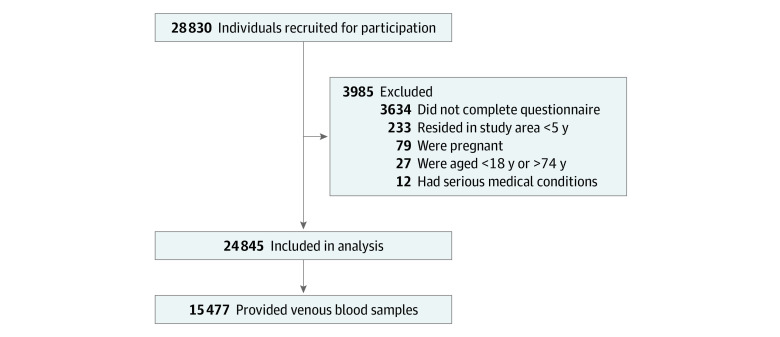
Participant Flowchart

### Cardiovascular Disease and Cardiometabolic Disorders

We assessed CVD status using a study questionnaire. A CVD case was defined as an affirmative response to the question: “Has a physician ever diagnosed you with myocardial infarction, heart failure, coronary heart disease, cerebral thrombosis, cerebral hemorrhage, cerebral embolism, or subarachnoid hemorrhage?”^45^

Using a standard mercuric-column sphygmomanometer, trained and certified nurses measured systolic blood pressure and diastolic blood pressure according to procedures recommended by the American Heart Association.^46^ We defined hypertension as mean systolic blood pressure higher than 140 mm Hg, mean diastolic blood pressure higher than 90 mm Hg, and/or reported receipt of antihypertensive medication in the 2 weeks before the interview.^47^

Using protocols recommended by the World Health Organization,^48^ we measured height and weight and calculated body mass index (BMI; calculated as weight in kilograms divided by height in meters squared) for each participant. Based on Asian criteria,^48^ obese status was defined as a BMI of 27.5 or higher, overweight status as a BMI of 23.0 to 27.5, and normal weight (including underweight) status as a BMI of less than 23.0.

After an overnight fast, peripheral venous blood samples were obtained. The levels of total cholesterol, triglycerides, low-density lipoprotein (LDL) cholesterol, and high-density lipoprotein (HDL) cholesterol were determined using a Hitachi Autoanalyzer, type 7170A (Hitachi). We defined hypercholesterolemia as a total cholesterol level of 240 mg/dL or higher, hypertriglyceridemia as a triglyceride level of 200 mg/dL or higher, low HDL cholesterol as an HDL cholesterol level of 40 mg/dL or higher, and high LDL cholesterol as an LDL cholesterol level of 160 mg/dL or higher.^49^

We performed a standard 75-g oral glucose tolerance test and obtained blood samples at 0 hours and 2 hours after glucose intake. We measured fasting and 2-hour glucose levels using an enzymatic colorimetric method. Type 2 diabetes was defined based on American Diabetes Association guidelines as a fasting blood glucose level of 126 mg/dL or higher (to convert to mmol/L, multiply by 0.0555), a 2-hour glucose level of 200 mg/dL or higher, or the reported receipt of antidiabetic medication.^50^

### Residential Greenness Levels

We applied 2 satellite-based vegetation indices, the normalized difference vegetation index (NDVI)^51^ and the soil-adjusted vegetation index (SAVI),^52^ to characterize residential greenness levels. Both indices are derived based on the difference of surface reflectance and absorbance in 2 vegetation-informative light bands, visible red and near-infrared. The NDVI is calculated by subtracting visible red light from near-infrared light and dividing the difference by the sum of near-infrared light and visible red light. For SAVI, a correction factor was added to minimize factors associated with the soil background. Therefore, the SAVI was calculated by subtracting visible red light from near-infrared light and dividing the difference by the sum of near-infrared light, visible red light, and the correction factor; the dividend was then multiplied by the sum of the correction factor and 1.0. The resulting NDVI and SAVI values were unitless and ranged from –1.0 to 1.0, with higher values indicating greener areas, negative values indicating bodies of water, and values close to 0 indicating barren areas.

The 2 indices were calculated using Landsat 5 Thematic Mapper (National Aeronautics and Space Administration) satellite images at a spatial resolution of 30 m by 30 m, which were obtained during the greenest month (ie, August, which has the highest level of growing vegetation) and the year closest to the collection of health data (ie, 2010). Residential greenness was defined as the mean NDVI and SAVI levels in circular buffers of 500 m and 1000 m around each community’s centroid. Greenness level was defined using the NDVI, with low greenness level defined as less than 0.40 m of vegetation per 500 m, and high greenness level defined as 0.40 m or more of vegetation per 500 m. All calculations were performed using ArcGIS, version 10.4 (Esri).

### Confounding and Mediating Factors

We developed a directed acyclic graph to select confounding variables and potential mediating factors using the DAGitty package, version 2.16.3 for R software (R Foundation for Statistical Computing) (eFigure 1 in the Supplement). The following confounding variables were retained: age, sex, ethnicity, annual mean household income, educational level, physical activity level, district-level gross domestic product,^39^ and particulate matter with an aerodynamic diameter of 2.5 μm or less.^53^

Cardiometabolic disorders were selected as potential mediating factors (eFigure 1 in the Supplement). We developed an additional directed acyclic graph to identify potential confounding variables of exposure-mediator associations (eFigure 2 in the Supplement) and mediator-outcome associations (eFigure 3 in the Supplement). We incorporated the common confounding variables (ie, age, sex, ethnicity, household income, educational level, physical activity level, district-level gross domestic product, and particulate matter with an aerodynamic diameter of 2.5 μm or less) into the mediation analysis.

### Statistical Analysis

We used generalized linear mixed models^54^ to assess the association between residential greenness levels and CVD prevalence, with communities considered a random effect. The effect estimates were expressed as odds ratios (ORs) and 95% CIs per interquartile range (IQR) increase in NDVI and SAVI levels. We estimated unadjusted models and main models that were adjusted for the confounding variables selected using the directed acyclic graph (including age, sex, ethnicity, household income, educational level, physical activity level, district-level gross domestic product, and particulate matter with an aerodynamic diameter of 2.5 μm or less) (eFigure 1 in the Supplement). Informed by findings from previous studies,^55,56^ we focused on a 500-m exposure buffer for the main analysis.

We also performed several sensitivity analyses. First, we estimated NDVI and SAVI levels in a larger exposure buffer of 1000 m to assess the impact of exposure misclassification. Second, we categorized NDVI and SAVI levels per 500 m into quartiles (for NDVI levels, quartile 1 comprised 0.18-0.23, quartile 2 comprised 0.24-0.29, quartile 3 comprised 0.30-0.40, and quartile 4 comprised 0.41-0.80; for SAVI levels, quartile 1 comprised 0.10-0.13, quartile 2 comprised 0.14-0.16, quartile 3 comprised 0.17-0.24, and quartile 4 comprised 0.25-0.48), and we used a natural spline-smoothing function to test for nonlinear associations. Third, we built models using sequential adjustments, and we adjusted the main models for alcohol consumption, cigarette smoking, physical activity level, controlled diet with low calories and low fat, consumption of sugar-sweetened soft drinks, and family history of CVD to assess their potential impact. We performed stratified analyses by age (<50 years vs ≥50 years), sex, annual household income (<10 000 yuan vs ≥10 000 yuan [$1 was equivalent to 6.84 yuan in 2009]), and educational level (<9 years vs ≥9 years) to examine whether the associations were consistent among different subpopulations.

We used a 3-way decomposition method to apportion the association between residential greenness and each cardiometabolic disorder with which the greenness level may have interacted with CVD prevalence. The 3 nonoverlapping components included (1) variables associated with greenness that were directly controlled for potential mediating factors (direct effect), (2) variables associated with additive interaction but not mediation (interactive effect), and (3) variables associated with mediation but not interaction (pure indirect effect) (eFigure 4 in the Supplement).^57^ Standard errors were calculated using the delta method.^58^ We also estimated the mediation effect of combined cardiometabolic disorders using the lavaan package, version 3.4.3 for R software (R Foundation for Statistical Computing). All other statistical analyses were performed using SAS software, version 9.4 (SAS Institute Inc). All tests were 2-tailed and paired, with a significance threshold of *P* < .05.

## Results

Among 24 845 adult participants, the mean (SD) age was 45.6 (13.3) years, and 12 661 participants (51.0%) were men (Table 1). Most of the participants (94.5%) were of Han ethnicity and had a high educational level (78.0% of participants had >9 years of education) and high household income (76.8% of participants had a mean household income of >10 000 yuan per year). A total of 1006 participants (4.1%) reported having a diagnosis of CVD. Participants with CVD were more likely to be male (743 participants [73.9%]), 50 years or older (749 participants [75.5%]), of Han ethnicity (980 participants [97.4%]), have an educational level of 9 years or less (836 participants [93.1%]), and have an annual household income of more than 10 000 yuan (640 participants [63.6%]) (eTable 3 in the Supplement). Participants living in communities with higher greenness levels were more likely to be female (3106 participants [59.4%]), younger than 50 years (3662 participants [70.0%]), have an educational level of 9 years or less (3932 participants [75.2%]), have an annual household income of more than 10 000 yuan (4193 participants [80.2%]), exercise less than 180 minutes per week (3547 participants [67.8%]), and live in communities with lower concentrations of particulate matter with an aerodynamic diameter of 2.5 μm or less (71 participants [30.5%]). The prevalence of cardiometabolic disorders ranged from 1333 participants (8.6%) with high LDL cholesterol to 15 459 participants (62.2%) with overweight or obese status.

**Table 1.** Participant Characteristics

Characteristic	No. (%)		
	Total	Residential greenness level	
		Low^a^	High^a^
Total participants	24 845 (100)	19 616 (79.0)	5229 (21.0)
Age, y			
<50	15 503 (62.4)	11 841 (60.4)	3662 (70.0)
≥50	9342 (37.6)	7775 (39.6)	1567 (30.0)
Sex			
Male	12 661 (51.0)	10 538 (53.7)	2123 (40.6)
Female	12 184 (49.0)	9078 (46.3)	3106 (59.4)
Ethnicity			
Han	23 470 (94.5)	18 552 (94.6)	4918 (94.1)
Other	1375 (5.5)	1064 (5.4)	311 (5.9)
Educational level, y			
≤9	19 370 (78.0)	15 438 (78.7)	3932 (75.2)
>9	5475 (22.0)	4178 (21.3)	1297 (24.8)
Annual household income, yuan^b^			
≤10 000	5761 (23.2)	4725 (24.1)	1036 (19.8)
>10 000	19 084 (76.8)	14 891 (75.9)	4193 (80.2)
Physical activity ≥180 min/wk			
Yes	7647 (30.8)	5965 (30.4)	1682 (32.2)
No	17 198 (69.2)	13 651 (69.6)	3547 (67.8)
District-level per capita GDP, median (IQR), yuan^b^	70 352 (47 639-100 423)	70 352 (47 639-100 423)	74 266 (25 561-100 423)
PM ≤ 2.5 μm/m^3^, median (IQR), μg/m^3^	73.00 (71.00-97.00)	72.82 (72.26-97.74)	70.97 (63.87-94.39)
Cardiometabolic disorder			
Hypertension	8657 (34.8)	7099 (36.2)	1558 (29.8)
Overweight/obese	15 459 (62.2)	12 473 (63.6)	2986 (57.1)
Type 2 diabetes	1694 (10.9)^c^	1429 (11.5)^d^	265 (8.8)^e^
Hypercholesterolemia	1717 (11.1)^c^	1456 (11.7)^d^	261 (8.7)^e^
Hypertriglyceridemia	3494 (22.6)^c^	2931 (23.5)^d^	563 (18.7)^e^
Low HDL cholesterol	2836 (18.3)^c^	2400 (19.3)^d^	436 (14.5)^e^
High LDL cholesterol	1333 (8.6)^c^	1147 (9.2)^d^	186 (6.2)^e^
Cardiovascular disease^f^	1006 (4.1)	871 (4.4)	135 (2.6)

Abbreviations: GDP, gross domestic product; HDL, high-density lipoprotein; IQR, interquartile range; LDL, low-density lipoprotein; PM, particulate matter.

^a^ Greenness level was based on normalized difference vegetation index values. Low greenness level was defined as less than 0.40 m of vegetation per 500 m, and high greenness level was defined as 0.40 m or more of vegetation per 500 m.

^b^ $1.00 was equivalent to 6.84 yuan in 2009.

^c^ Based on 15 477 participants.

^d^ Based on 12 470 participants.

^e^ Based on 3007 participants.

^f^ A total of 417 participants had heart disease, 529 participants had stroke, and 60 participants had heart disease and stroke.

Greenness levels varied across the communities. For instance, NDVI levels per 500 m ranged from 0.18 to 0.80, with a median of 0.29 (IQR, 0.23-0.40), and SAVI levels per 500 m ranged from 0.10 to 0.48, with a median of 0.16 (IQR, 0.13-0.24) (eTable 4 in the Supplement). In addition, NDVI levels were consistent with SAVI levels. For example, the lowest Spearman correlation coefficient was 0.88 for the correlation between NDVI levels per 500 m and SAVI levels per 1000 m, and the highest Spearman correlation coefficient was 0.98 for the correlation between NDVI levels per 500 m and SAVI levels per 500 m (eTable 4 in the Supplement).

In the adjusted models, an IQR increase in both NDVI and SAVI levels per 500 m was associated with a lower likelihood of CVD prevalence, with ORs of 0.73 (95% CI, 0.65-0.83; *P* < .001) for NDVI levels and 0.74 (95% CI, 0.66-0.84; *P* < .001) for SAVI levels (Table 2). Similar results were observed for NDVI levels per 1000 m (OR, 0.79; 95% CI, 0.71-0.89; *P* < .001) and SAVI levels per 1000 m (OR, 0.78; 95% CI, 0.69-0.87; *P* < .001). The effect estimates were similar to those of the main models when we adjusted for alcohol use (for NDVI: OR, 0.74; 95% CI, 0.65-0.83; *P* < .001; for SAVI: OR, 0.74; 95% CI, 0.66-0.84; *P* < .001), cigarette smoking (for NDVI: OR, 0.73; 95% CI, 0.64-0.86; *P* < .001; for SAVI: OR, 0.73; 95% CI, 0.65-0.83; *P* < .001), low-calorie and low-fat diet (for NDVI: OR, 0.73; 95% CI, 0.65-0.82; *P* < .001; for SAVI: OR, 0.74; 95% CI, 0.65-0.83; *P* < .001), consumption of sugar-sweetened soft drinks (for NDVI: OR, 0.73; 95% CI, 0.64-0.82; *P* < .001; for SAVI: OR, 0.73; 95% CI, 0.65-0.83; *P* < .001), and family history of CVD (for NDVI: OR, 0.73; 95% CI, 0.65-0.82; *P* < .001; for SAVI: OR, 0.74; 95% CI, 0.65-0.83; *P* < .001) and when we incorporated sequential adjustments (eg, in model 1, which was adjusted for age, sex, ethnicity, and community size, the OR for NDVI was 0.71; 95% CI, 0.63-0.80; *P* < .001;for SAVI, 0.72; 95% CI, 0.64-0.81; *P* < .001) (eTable 5 in the Supplement).

**Table 2.** Association Between Residential Greenness Measures and Cardiovascular Disease Prevalence

Greenness measure^a^	Unadjusted OR (95% CI)	*P* value	Adjusted OR (95% CI)^b^	*P* value
NDVI per 500 m	0.57 (0.51-0.64)	<.001	0.73 (0.65-0.83)	<.001
NDVI per 1000 m	0.63 (0.57-0.70)	<.001	0.79 (0.71-0.89)	<.001
SAVI per 500 m	0.58 (0.51-0.65)	<.001	0.74 (0.66-0.84)	<.001
SAVI per 1000 m	0.62 (0.56-0.69)	<.001	0.78 (0.69-0.87)	<.001

Abbreviations: NDVI, normalized difference vegetation index; OR, odds ratio; SAVI, soil-adjusted vegetation index.

^a^ Greenness level per interquartile range increase.

^b^ Adjusted for age, sex, ethnicity, household income, educational level, district-level gross domestic product, physical activity level, and air pollution level.

We found similar linear dose-response patterns when using NDVI and SAVI levels per 500 m categorized into quartiles (eg, for quartile 2, the adjusted OR for NDVI was 1.03; 95% CI, 0.86-1.23; for quartile 3, the adjusted OR for NDVI was 0.66; 95% CI, 0.55-0.80; for quartile 4, the adjusted OR for NDVI was 0.63; 95% CI, 0.50-0.79; with *P* < .001 for trend) (eTable 6 in the Supplement) and when using a restrictive cubic spline analysis (for NDVI, *P* for nonlinear association = 0.74; for SAVI, *P* for nonlinear association = 0.62) (eFigure 5 in the Supplement). We found no significant interaction by age, sex, household income, or educational level (eTable 7 in the Supplement).

The mediation analyses examining the association between NDVI level per 500 m and CVD prevalence indicated mediation effects of 4.5% (95% CI, 0.6%-8.3%; *P* = .02) for hypertension, 3.1% (95% CI, 1.1%-5.1%; *P* = .002) for overweight or obese status, 4.1% (95% CI, 0.7%-7.4%; *P* = .02) for type 2 diabetes, 12.7% (95% CI, 4.6%-20.8%; *P* = .002) for hypercholesterolemia, 8.7% (95% CI, 2.9%-14.4%; *P* = .003) for hypertriglyceridemia, and 11.1% (95% CI, 3.8%-18.4%; *P* = .003) for high LDL cholesterol. No significant mediation effect was found for low HDL cholesterol (1.9%; 95% CI, −0.3% to 4.0%; *P* = .09) (Table 3). Combined, these cardiometabolic disorders jointly mediated 21.2% (95% CI, 6.4%-35.9%; *P* = .004) of the association between NDVI level per 500 m and CVD prevalence (eTable 8 in the Supplement). Similar results were obtained when we used SAVI level per 500 m as the exposure measure, with a significant mediation effect for all cardiometabolic disorders (eg, for hypercholesterolemia, 13.0%; 95% CI, 5.0%-21.0%; *P* = .001) with the exception of low HDL cholesterol (2.0%; 95% CI, −0.2% to 4.2%; *P* = .08). No significant interactive effect was found between residential greenness and any cardiometabolic disorder (Table 3).

**Table 3.** Association Between Residential Greenness, Cardiometabolic Disorders, and Cardiovascular Disease Prevalence

Exposure	Potential mediator	Participants, No.	Decomposition method, % (95% CI)^a^					
			Mediation effect	*P* value	Interactive effect	*P* value	Direct effect	*P* value
NDVI per 500 m	Hypertension	24 845	4.5 (0.6 to 8.3)	.02	7.0 (−18.4 to 32.4)	.59	88.5 (62.9 to 114.1)	<.001
	Type 2 diabetes	15 477	4.1 (0.7 to 7.4)	.02	−5.5 (−19.4 to 8.5)	.44	101.4 (86.4 to 116.4)	<.001
	Overweight/obese	24 845	3.1 (1.1 to 5.1)	.002	38.1 (−6.6 to 82.7)	.10	58.8 (13.2 to 104.4)	.01
	Hypercholesterolemia	15 477	12.7 (4.6 to 20.8)	.002	−4.8 (−26.9 to 17.2)	.67	92.1 (68.9 to 125.4)	<.001
	Hypertriglyceridemia	15 477	8.7 (2.9 to 14.4)	.003	−0.8 (−32.4 to 30.7)	.96	92.2 (59.6 to 124.7)	<.001
	Low HDL cholesterol	15 477	1.9 (−0.3 to 4.0)	.09	−27.7 (−58.9 to 3.4)	.08	125.9 (94.7 to 157.1)	<.001
	High LDL cholesterol	15 477	11.1 (3.8 to 18.4)	.003	−7.2 (−25.3 to 10.8)	.43	96.1 (76.9 to 115.4)	<.001
SAVI per 500 m	Hypertension	24 845	5.1 (1.2 to 9.0)	.01	9.3 (−15.3 to 33.9)	.46	85.6 (60.6 to 110.6)	<.001
	Type 2 diabetes	15 477	4.3 (0.9 to 7.7)	.01	−3.3 (−16.4 to 9.7)	.62	99.0 (84.7 to 113.4)	<.001
	Overweight/obese	24 845	3.0 (1.1 to 5.0)	.002	41.9 (−2.8 to 86.6)	.07	55.0 (9.4 to 100.7)	.02
	Hypercholesterolemia	15 477	13.0 (5.0 to 21.0)	.001	−1.1 (−21.4 to 19.3)	.92	88.0 (65.7 to 110.3)	<.001
	Hypertriglyceridemia	15 477	8.1 (2.8 to 13.4)	.003	2.3 (−26.7 to 31.3)	.88	89.6 (59.4 to 119.)	<.001
	Low HDL cholesterol	15 477	2.0 (−0.2 to 4.2)	.08	−22.0 (−50.4 to 6.4)	.13	120.0 (91.3 to 148.7)	<.001
	High LDL cholesterol	15 477	11.6 (4.2 to 18.9)	.002	−4.1 (−20.7 to 12.5)	.63	92.5 (74.0 to 111.1)	<.001

Abbreviations: HDL, high-density lipoprotein; LDL, low-density lipoprotein; NDVI, normalized difference vegetation index; SAVI, soil-adjusted vegetation index.

^a^ Adjusted for age, sex, ethnicity, household income, educational level, district-level gross domestic product, physical activity level, and air pollution level.

## Discussion

In this study, we found that participants living in greener communities had a lower likelihood of receiving a diagnosis of CVD. The association between residential greenness and CVD was robust in a series of sensitivity analyses. The presence of cardiometabolic disorders (with the exception of low HDL cholesterol) partially mediated the association between residential greenness levels and CVD prevalence.

Many previous studies have documented an association between greenness levels and CVD (eTable 1 in the Supplement),^5,6,7,8,9,10,11,12,13,14,15,16,17,18,19,20,21,22,23,24,25,26,27,28,29,30,31,32,33,34,35,36,37^ which is consistent with our current findings. For example, a cross-sectional study of 11 404 Australian adults reported that higher neighborhood greenness levels (measured as the mean NDVI level per 1600 m) and variability (measured as the SD of the NDVI level per 1600 m) were associated with a lower prevalence of heart disease or stroke.^13^ Another cross-sectional study of Chinese adults reported a substantial reduction in the likelihood of coronary heart disease and stroke among populations residing in areas with higher NDVI levels.^30^ Similar results were reported by cross-sectional studies from the US,^36^ Israel,^28^ New Zealand,^14^ and Brazil.^23^

Although our study was cross-sectional and used NDVI levels to estimate exposure, our findings may support those from 2 prospective cohort studies^15,32^ that used different green space measures and 1 natural experiment study.^18^ The first cohort study followed up 5112 Lithuanian adults for 4.4 years. The investigators found that a greater distance from green spaces was associated with an increased risk of CVD.^15^ The second cohort study found that a larger tree canopy percentage was associated with a lower risk of incident CVD among 46 786 Australian adults.^32^ In the natural experiment study, Donovan et al^21^ analyzed longitudinal data from 156 146 women and examined the association between the loss of trees owing to an invasive forest pest and incident CVD. They reported that women living in areas with greater tree loss had an increased risk of CVD.^21^

We observed that all of the explored cardiometabolic disorders (with the exception of low HDL cholesterol) substantially mediated the association between residential greenness and CVD prevalence, indicating that the disorders might be involved in the pathways by which greenness is associated with CVD. Our findings were consistent with those of a US study,^36^ which found that the associations between NDVI levels and CVD were attenuated when regression models were adjusted for the presence of hypertension, type 2 diabetes, and dyslipidemia. Partially consistent with our findings, another study reported that the association of myocardial infarction with NDVI levels was partially mediated by the presence of dyslipidemia and type 2 diabetes but not hypertension.^28^ Combined with our findings, the data extended our understanding of potential biological mechanisms underlying the association between residential greenness and CVD prevalence. Because cardiometabolic disorders are reported to be precursors to clinical CVD, the results of our mediation analysis suggest that future research and health care decisions that focus on the association of greenness with these earlier disorders may be useful for reducing future CVD events.

An earlier study suggested several pathways, such as reduction in air pollution and heat, encouragement of physical activity, increases in social cohesion, and decreases in mental fatigue and stress,^59^ to explain the association between greenness and health. However, accumulating data do not provide support for an association between substantial air pollution reductions or increases in physical activity and a greater number of urban greenness areas.^60,61^ Because data on social cohesion and mental health were not available for our study, we did not investigate those potential factors. Future studies are needed to explore potential mechanisms, preferably through the inclusion of more mediators and the performance of serial mediation tests.

To our knowledge, this is one of a few epidemiologic studies to report an association between residential greenness and CVD in a low- or middle-income country. Our results may have public health importance, especially considering the fact that many low-income countries are experiencing rapid urbanization and subsequent decreases in greenness. A major strength of our study is its large population-based sample, which was randomly selected from communities across 3 different cities, and its high response rate, which minimized selection bias and provided sufficient statistical power to detect modest effects. In addition, we used 2 greenness exposure measures and 2 different exposure buffers, and we adjusted for a parsimonious yet comprehensive set of covariates to control confounding without overadjustment. Furthermore, we performed mediation analyses using a 3-way decomposition method, which incorporates interactive effects and typically provides greater validity and higher statistical power than traditional approaches.^57^

### Limitations

This study has several limitations. First, the cross-sectional design did not allow us to assess temporality between greenness exposure and CVD, although the likelihood of reverse-association is low. In addition, the cross-sectional design may have overestimated the mediation effects.^62^ Second, we assessed greenness exposure based on communities and not individuals, which might have produced measurement error (ie, Berkson error^63^). Although this error did not bias our effect estimates, it could have reduced statistical power. In addition, we have not specifically collected data on limited mobility. Third, although we objectively measured greenness exposure using NDVI and SAVI levels, these 2 measures reflect general vegetation levels and cannot differentiate the structure, type, and quality of greenness. Thus, we were unable to examine aspects of greenness that are most relevant to CVD prevalence.

Fourth, data on antidyslipidemia medication receipt were not available and were not used to define dyslipidemia, which might have reduced the prevalence of dyslipidemia and biased the results of our mediation analysis. Fifth, CVD diagnosis and several potential confounding variables were self-reported; therefore, false-positive findings and recall bias are possible. In addition, we assessed socioeconomic status using a limited number of variables, and other environmental factors, such as noise, walkability, and air temperature, were not considered in our model; thus, our results might have been affected by residual confounding.

## Conclusions

Higher community greenness levels were associated with a lower likelihood of CVD prevalence in an industrial and highly urbanized setting within China. The association might be partly mediated by the presence of cardiometabolic disorders. Although the findings require confirmation in longitudinal studies, they contribute to the limited data available to policy makers who are interested in designing population-level interventions to mitigate the prevalence of CVD in China and other low- and middle-income countries.

## Notes

Supplement. **eTable 1.** Previous Human Epidemiological Studies of Greenness Exposure and CVD Mortality and Morbidity **eTable 2.** Characteristics of Study Participants With and Without Blood Sampling **eTable 3.** Main Characteristics of Study Participants With and Without CVD **eTable 4.** Distributions and Intercorrelations (Spearman Correlation Coefficients) of NDVI and SAVI **eTable 5.** Sensitivity Analyses of Associations Between Greenness Measures and CVD Prevalence Using Different Adjusted Models **eTable 6.** Associations Between Quartiles of Greenness Measures and CVD Prevalence **eTable 7.** Associations of Greenness With CVD Prevalence by Age, Sex, and Annual Household Income **eTable 8.** Mediation of Association Between Greenness and CVD Prevalence by Combined Cardiometabolic Disorders **eFigure 1.** Directed Acyclic Graph for the Association Between Greenness and CVD **eFigure 2.** Directed Acyclic Graph for the Association Between Greenness and Cardiometabolic Disorders **eFigure 3.** Directed Acyclic Graph for the Association Between Cardiometabolic Disorders and CVD **eFigure 4.** Three-Way Decomposition Encompassing Decompositions for Both Mediation and Interaction **eFigure 5.** Dose-Response Curves for Greenness Levels With CVD Prevalence **eReferences**
