# Supplementary material for: Association Between Residential Greenness, Cardiometabolic Disorders, and Cardiovascular Disease Among Adults in China
Source: JAMA Netw Open. 2020 Sep 21;3(9):e2017507. doi: 10.1001/jamanetworkopen.2020.17507 (PMC7506516; doi:10.1001/jamanetworkopen.2020.17507)
Supplement: Supplement. — eTable 1. Previous Human Epidemiological Studies of Greenness Exposure and CVD Mortality and Morbidity eTable 2. Characteristics of Study Participants With and Without Blood Sampling eTable 3. Main Characteristics of Study Participants With and Without CVD eTable 4. Distributions and Intercorrelations (Spearman Correlation Coefficients) of NDVI and SAVI eTable 5. Sensitivity Analyses of Associations Between Greenness Measures and CVD Prevalence Using Different Adjusted Models eTable 6. Associations Between Quartiles of Greenness Measures and CVD Prevalence eTable 7. Associations of Greenness With CVD Prevalence by Age, Sex, and Annual Household Income eTable 8. Mediation of Association Between Greenness and CVD Prevalence by Combined Cardiometabolic Disorders eFigure 1. Directed Acyclic Graph for the Association Between Greenness and CVD eFigure 2. Directed Acyclic Graph for the Association Between Greenness and Cardiometabolic Disorders eFigure 3. Directed Acyclic Graph for the Association Between Cardiometabolic Disorders and CVD eFigure 4. Three-Way Decomposition Encompassing Decompositions for Both Mediation and Interaction eFigure 5. Dose-Response Curves for Greenness Levels With CVD Prevalence eReferences [file jamanetwopen-e2017507-s001.pdf]

Supplementary Online Content

Yang B-Y, Hu L-W, Jalaludin B, et al. Association between residential greenness, cardiometabolic disorders, and cardiovascular disease among adults in China. *JAMA Netw Open*. 2020;3(9):e2017507. doi:10.1001/jamanetworkopen.2020.17507

eTable 1. Previous Human Epidemiological Studies of Greenness Exposure and CVD Mortality and Morbidity

eTable 2. Characteristics of Study Participants With and Without Blood Sampling

eTable 3. Main Characteristics of Study Participants With and Without CVD

eTable 4. Distributions and Intercorrelations (Spearman Correlation Coefficients) of NDVI and SAVI

eTable 5. Sensitivity Analyses of Associations Between Greenness Measures and CVD Prevalence Using Different Adjusted Models

eTable 6. Associations Between Quartiles of Greenness Measures and CVD Prevalence

eTable 7. Associations of Greenness With CVD Prevalence by Age, Sex, and Annual Household Income

eTable 8. Mediation of Association Between Greenness and CVD Prevalence by Combined Cardiometabolic Disorders

eFigure 1. Directed Acyclic Graph for the Association Between Greenness and CVD

eFigure 2. Directed Acyclic Graph for the Association Between Greenness and Cardiometabolic Disorders

eFigure 3. Directed Acyclic Graph for the Association Between Cardiometabolic Disorders and CVD

eFigure 4. Three-Way Decomposition Encompassing Decompositions for Both Mediation and Interaction

eFigure 5. Dose-Response Curves for Greenness Levels With CVD Prevalence

eReferences

This supplementary material has been provided by the authors to give readers additional information about their work.

© 2020 Yang B-Y et al. *JAMA Network Open*.

eTable 1. Previous Human Epidemiological Studies of Greenness Exposure and CVD Mortality and Morbidity

Author (Year)	Sample size	Study design	Study setting	Exposure	Outcome	Main findings
Hu et al. (2008)[1]	Not reported	Cross-sectional	USA	Distance to and frequency of visiting green space; satellite-derived greenness	Stroke mortality	High risk of stroke mortality was found in areas with low level of exposure to green space
Mitchell et al. (2008)[2]	40,813,236	Cross-sectional	England	Percentage of greenspace	CVD mortality	Lower mortality incidence rate ratio was associated with higher levels of green space
Mass et al. (2009)[3]	345,143	Cross-sectional	Dutch	Percentage of green space	CVD prevalence	More green space within a 1 km radius around the postal code coordinates was associated with lower odds of CVD prevalence
Richardson et al. (2010)[4]	1,546,405	Ecological	New Zealand	Percent coverage of green space types	CVD mortality	No significant association was observed for usable or total green space with CVD mortality
Dominguez-Berjon et al. (2010)[5]	5,423,384	Cross-sectional	Spain	Dearth of green space (surveyed using questionnaire)	CVD mortality	Lack of green space was associated with higher risk of death due to ischemic heart disease in men, and with death due to cerebrovascular disease in both men and women
Coutts et al. (2010)[6]	Not reported	Cross-sectional	USA	Amount of greenspace within defined distance	CVD mortality	The amount of greenspace within defined distances of census tract in each county was associated with CVD mortality
Richardson et al. (2010)[7]	28.6 million	Cross-sectional	UK	Proportion of greenspace by area	CVD mortality	CVD mortality rates decreased with increasing green space only in males.
Richardson et al. (2012)[8]	43 million	Cross-sectional	USA	Population weighted green space coverage	CVD mortality	No association was observed between green space coverage and CVD mortality
Pereira et al. (2012)[9]	11,404	Cross-sectional	Australia	NDVI	CVD prevalence	Levels and variability of greenness were inversely associated with

© 2020 Yang B-Y et al. *JAMA Network Open*.

						prevalence of coronary heart disease and stroke.
Donovan et al. (2013)[10]	21,080	Natural experiment	USA	The emerald ash borer, an invasive forest pest	CVD mortality	Loss of trees (i.e., reduction in greenness) to the emerald ash borer increased mortality related to cardiovascular illness.
Richardson et al. (2013)[11]	8157	Cross-sectional	New Zealand	Proportion of green space within each census area unit	CVD prevalence	CVD risk was reduced in all neighborhoods with >15% greenspace availability (e.g. OR 0.80, 95% CI, 0.64-0.99 for those with 33-70% green space).
Tamosiunas et al. (2014)[12]	5112	Cohort	Lithuania	Distance to the nearest green space	CVD incidence and mortality	Distance to green space was significantly associated with fatal and non-fatal CVD
Lzchowycz et al. (2014)[13]	165,424	Cross-sectional	England	Percentage of greenspace	CVD mortality	Decreased premature circulatory mortality was found in greener areas, especially in those most deprived group.
Wilker et al. (2014)[14]	1763	Cohort	USA	NDVI	CVD mortality	Compared with stroke patients living in the lowest quartile of green space, those living in the highest quartile of green space had lower risk of death
Bixby et al. (2015)[15]	≥100,000	Cross-sectional	England	Proportion of city area covered by “green” land	CVD mortality	No significant association was observed between green space proportion and CVD mortality
Chum et al. (2015)[16]	2411	Cross-sectional	Canada	Proportion of parks and recreational areas within each census tract	CVD prevalence	No significant association was observed between proportion of parks and recreational areas and CVD prevalence.
Donovan et al. (2015)[17]	156,146	Cohort	USA	The emerald ash borer	CVD incidence	Women living in a county infested with emerald ash borer had an increased risk of CVD
James et al. (2016)[18]	108,630	Cohort	USA	NDVI	CVD mortality	Higher levels of green vegetation were associated with decreased mortality
Massa et al. (2016)[19]	1333	Cross-sectional	Brazil	Total green area per square meter	CVD prevalence	In comparison to participants living with the low green space levels, those in higher levels were

© 2020 Yang B-Y et al. *JAMA Network Open*.

						significantly less likely to report having a CVD.
Ngom et al. (2016)[20]	3,920,000	Cross-sectional	Canada	The nearest distance to green spaces	CVD prevalence	Among the various green space types, only green spaces with sport facilities showed a significant relationship with diabetes and cerebrovascular disease morbidity
Picavet et al. (2016)[21]	12,546	Cross-sectional	Netherlands	Percentage of green space	CVD prevalence	No significant association was observed between percentage of green space and CVD prevalence
Vienneau et al. (2017)[22]	4.2 million	Cohort	Switzerland	NDVI; land use green exposure	CVD mortality	Higher NDVI levels were associated with lower CVD mortality
Wang et al. (2017)[23]	3544	Cohort	China	NDVI	CVD mortality	A 10% increase in coverage of green space was significantly associated with a reduction in all-cause mortality
Yitshak-Sade et al.(2017)[24]	23,110	Cross-sectional	Israel	NDVI	CVD prevalence	NDVI was associated with myocardial infarction
Crouse et al. (2017)[25]	1,265,000	Cohort	Canada	NDVI	CVD prevalence and mortality	Per 0.15-unit increase in NDVI-250m was significantly associated with 0.960 (95% CI: 0.943-0.976), 0.949 (95% CI: 0.927-0.972), and 1.042 (95% CI: 0.963-1.047)-fold risk of cardiovascular disease, ischemic heart disease, and cerebrovascular disease, respectively.
Jia et al. (2018)[26]	1944	Cross-sectional	China	NDVI	CVD prevalence	Compared to the participants with low NDVI level, participants with moderate to high levels of NDVI had a 75% and 45% reduced odds of coronary heart disease and stroke, respectively
Silveira et al. (2018)[27]	6,230,446	Cross-sectional	Brazil	NDVI	CVD mortality	Mortality rates for CVD are inversely associated with greenness exposure
Astell-Burt et al. (2019)[28]	46,786	Cohort	Australia	Percentage of green space and tree canopy	CVD incidence	Larger percentage of tree canopy, but not green space, was associated with lower odds of incident CVD.

© 2020 Yang B-Y et al. *JAMA Network Open*.

Orioli et al. (2019)[29]	1,263,721	Cohort	Italy	NDVI and LAI	CVD mortality	Residential greenness, expressed as NDVI and LAI, was inversely associated with stroke incidence and cardiovascular mortality.
Kim et al. (2019)[30]	73 districts with an average population per district of 317,869	Time-series	Korea	NDVI	CVD mortality	High level of greenness was associated with decreased risk of CVD-related mortality
Servadio et al. (2019)[31]	169 census tracts	Cross-sectional	USA	Park access and tree canopy cover	CVD prevalence	Greater percent tree canopy cover and green space access were associated with higher prevalence of coronary heart disease and stroke.
Wang et al. (2019)[32]	249,405	Cross-sectional	USA	NDVI	CVD prevalence	Compared with the lowest tertile of greenness, the highest tertile of greenness was associated with reduced odds of acute myocardial infarction by 25%, ischaemic heart disease by 20%, heart failure by 16%, and atrial fibrillation by 6%. Additional adjustment for biological risk factors attenuated the associations.
Zijlema et al. (2019)[33]	9218	Cohort	Australia	NDVI, number and size of parks and nature space	CVD mortality	Access to natural spaces was associated with decreased mortality

Abbreviations: CI, confidence interval; CVD, cardiovascular disease; LAI, the leaf area index; NDVI, the normalized difference vegetation index; NP, not reported; OR, odds ratio; USA, the United States of America.

© 2020 Yang B-Y et al. *JAMA Network Open*.

eTable 2. Characteristics of Study Participants With and Without Blood Sampling

Characteristic	Participants with blood sampling (n = 15 477)	Participants without blood sampling (n = 9368)
Age (years, mean ± SD)	45.0 ± 13.5	45.7 ± 13.0
Gender, No. (%)		
Men	8156 (52.7)	4505 (48.1)
Women	7321 (47.3)	4863 (51.9)
Ethnicity, No. (%)		
Han	14 554 (94.0)	8916 (95.2)
Others	923 (6.0)	452 (4.8)
Education, No. (%)		
≤ 9 years	11 898 (76.9)	7472 (79.8)
> 9 years	3579 (23.1)	1896 (20.2)
Annual household income, No. (%)		
≤10 000 Yuan	3144 (20.3)	2617 (27.9)
>10 000 Yuan	12 333 (79.7)	6751 (72.1)

Abbreviations: SD, standard deviation.

© 2020 Yang B-Y et al. *JAMA Network Open*.

eTable 3. Main Characteristics of Study Participants With and Without CVD (N = 24 845)

**Characteristic**	**CVD (n = 1006)[a]**	**Non-CVD (n = 23 839)**	***P* value**
Age, No.(%)			<.001
<50 years	257 (25.5)	15 246 (64.0)	
≥50 years	749 (75.5)	8593 (36.0)	
Gender, No.(%)			<.001
Men	743 (73.9)	11 918 (50.0)	
Women	263 (26.1)	11 921 (50.0)	
Ethnicity, No.(%)			<.001
Han	980 (97.4)	22 490 (94.3)	
Others	26 (2.6)	1349 (5.7)	
Education, No.(%)			<.001
≤ 9 years	836 (93.1)	18 534 (77.8)	
> 9 years	170 (16.9)	5305 (22.3)	
Annual household income, No.(%)			<.001
≤10 000 Yuan	366 (36.4)	5395 (22.6)	
>10 000 Yuan	640 (63.6)	18 444 (77.4)	

Abbreviations: CVD, cardiovascular disease.
[a]CVD patients included 417 participants with heart disease, 529 with stroke, and 60 with both.

© 2020 Yang B-Y et al. *JAMA Network Open*.

eTable 4. Distributions and Intercorrelations (Spearman Correlation Coefficients) of NDVI and SAVI

	**Median (IQR)**	**Min**	**Max**	**$NDVI_{500\ m}$**	**$NDVI_{1000\ m}$**	**$SAVI_{500\ m}$**	**$SAVI_{1000\ m}$**
$NDVI_{500\ -m}$	0.29 (0.17)	0.18	0.80	1	0.90[a]	0.98[a]	0.88[a]
$NDVI_{1000\ -m}$	0.31 (0.15)	0.20	0.75		1	0.88[a]	0.96[a]
$SAVI_{500-\ m}$	0.16 (0.11)	0.10	0.48			1	0.90[a]
$SAVI_{1000\ -m}$	0.17 (0.10)	0.11	0.45				1

Abbreviations: IQR, interquartile range (computed by subtracting the 1st quartile from the 3rd quartile); max, maximum; min, minimum; NDVI, normalized difference vegetation index; SAVI, soil adjusted vegetation index.
[a]Statistically significant correlation (*P* < .05).

© 2020 Yang B-Y et al. *JAMA Network Open*.

eTable 5. Sensitivity Analyses of Associations Between Greenness Measures and CVD Prevalence Using Different Adjusted Models (N = 24 845)[a]

	$NDVI_{500\text{-}m}$			$SAVI_{500\text{-}m}$	
**Model**	**OR (95% CI)**	***P* value**		**OR (95% CI)**	***P* value**
Main model[b]	0.73 (0.65, 0.83)	<.0001		0.74 (0.66, 0.84)	<.0001
Main model[b] + alcohol drinking	0.74 (0.65, 0.83)	<.0001		0.74 (0.66, 0.84)	<.0001
Main model[b] + cigarette smoking	0.73 (0.64, 0.86)	<.0001		0.73 (0.65, 0.83)	<.0001
Main model[b] + low-calorie and low-fat diet	0.73 (0.65, 0.82)	<.0001		0.74 (0.65, 0.83)	<.0001
Main model[b] + sugar-sweetened soft drinks	0.73 (0.64, 0.82)	<.0001		0.73 (0.65, 0.83)	<.0001
Main model[b] + family history of CVD	0.73 (0.65, 0.82)	<.0001		0.74 (0.65, 0.83)	<.0001
Model 1[c]	0.71 (0.63, 0.80)	<.0001		0.72 (0.64, 0.81)	<.0001
Model 2[d]	0.71 (0.63, 0.80)	<.0001		0.71 (0.63, 0.81)	<.0001
Model 3[e]	0.70 (0.62, 0.79)	<.0001		0.70 (0.62, 0.79)	<.0001

Abbreviations: CI, confidence interval; CVD, cardiovascular disease; NDVI, normalized difference vegetation index; OR, odds ratio; SAVI, soil adjusted vegetation index

[a]Greenness per IQR Increase in $NDVI_{500\text{-}m}$ and $SAVI_{500\text{-}m}$.

[b]Adjusted for age, gender, ethnicity, household income, education, district-level of gross domestic product, physical activity, and air pollution ($PM_{2.5}$).

[c]Adjusted for age, gender, ethnicity, and community size

[d]Adjusted for age, gender, ethnicity, community size, education, household income, and district-level of gross domestic product

[e]Adjusted for age, gender, ethnicity, community size, education, household income, and district-level of gross domestic product, smoking status, physical activity level, low-calorie and low-fat diet, sugar-sweetened soft drinks, alcohol drinking, and family history of CVD.

© 2020 Yang B-Y et al. *JAMA Network Open*.

eTable 6. Associations Between Quartiles of Greenness Measures and CVD Prevalence (N = 24 845)

Greenness measure	Adjusted OR (95% CI)[a]	*P* value for trend
$NDVI_{500\text{-}m}$		<.0001
Q_1	Referent	
Q_2	1.03 (0.86, 1.23)	
Q_3	0.66 (0.55, 0.80)	
Q_4	0.63 (0.50, 0.79)	
$SAVI_{500\text{-}m}$		<.0001
Q_1	Referent	
Q_2	0.72 (0.61, 0.86)	
Q_3	0.55 (0.45, 0.67)	
Q_4	0.58 (0.48, 0.71)	

Abbreviations: CI, confidence interval; CVD, cardiovascular disease; NDVI, normalized difference vegetation index; OR, odds ratio; SAVI, soil adjusted vegetation index

[a]Adjusted for age, gender, ethnicity, household income, education, district-level of gross domestic product, physical activity, and air pollution.

© 2020 Yang B-Y et al. *JAMA Network Open*.

eTable 7. Associations of Greenness With CVD Prevalence by Age, Sex, and Annual Household Income (N = 24 845)[a]

	$NDVI_{500\text{-}m}$			$SAVI_{500\text{-}m}$	
**Subgroup**	**OR (95% CI)[b]**	$P_{interaction}$		**OR (95% CI)[b]**	$P_{interaction}$
Age		.39			.41
<50 years	0.71 (0.58 to 0.86)			0.71 (0.58 to 0.87)	
≥50 years	0.78 (0.68 to 0.90)			0.79 (0.68 to 0.91)	
Gender		.83			.81
Men	0.76 (0.66 to 0.88)			0.77 (0.66 to 0.89)	
Women	0.74 (0.61 to 0.90)			0.75 (0.61 to 0.91)	
Annual household income		.32			.33
<10 000 Yuan	0.82 (0.66 to 1.01)			0.83 (0.67 to 1.02)	
≥10 000 Yuan	0.73 (0.63 to 0.83)			0.73 (0.64 to 0.84)	
Education		.06			.09
<9 years	0.79 (0.69 to 0.90)			0.80 (0.70 to 0.91)	
≥9 years	0.60 (0.45 to 0.79)			0.61 (0.46 to 0.81)	

Abbreviations: CI, confidence interval; CVD, cardiovascular disease; IQR, interquartile range; NDVI, normalized difference vegetation index; OR, odds ratio; SAVI, soil adjusted vegetation index.

[a]Greenness per IQR in in $NDVI_{500\text{-}m}$ and $NDVI_{500\text{-}m}$.

[b]Adjusted for age, sex, ethnicity, household income, education, district-level of gross domestic product, physical activity, and air pollution ($PM_{2.5}$), except the variable that were stratified.

© 2020 Yang B-Y et al. *JAMA Network Open*.

eTable 8. Mediation of Association Between Greenness and CVD Prevalence by Combined Cardiometabolic Disorders[a]

	Indirect effect			Direct effect		
	**% (95% CI)[b]**	***P* value**		**% (95% CI)[b]**	***P* value**	
$NDVI_{500\text{-}m}$	21.2 (6.4 to 35.9)	.004		78.8 (64.1 to 93.4)	<.001	
$SAVI_{500\text{-}m}$	21.3 (7.4 to 35.2)	.003		78.7 (64.8 to 92.6)	<.001	

Abbreviations: CI, confidence interval; CVD, cardiovascular disease; NDVI, normalized difference vegetation index; SAVI, soil adjusted vegetation index.
[a]Cardiovascular disorders included hypertension, diabetes, overweight/obesity, hypercholesterolemia, hypertriglyceridemia, and high LDL-C, which are individually significantly mediated the association between greenness and CVD prevalence.
[b]Adjusted for age, gender, education, ethnicity, household income, physical activity, district-level gross domestic product, and air pollution ($PM_{2.5}$).

© 2020 Yang B-Y et al. *JAMA Network Open*.

eFigure 1. Directed Acyclic Graph for the Association Between Greenness and CVD

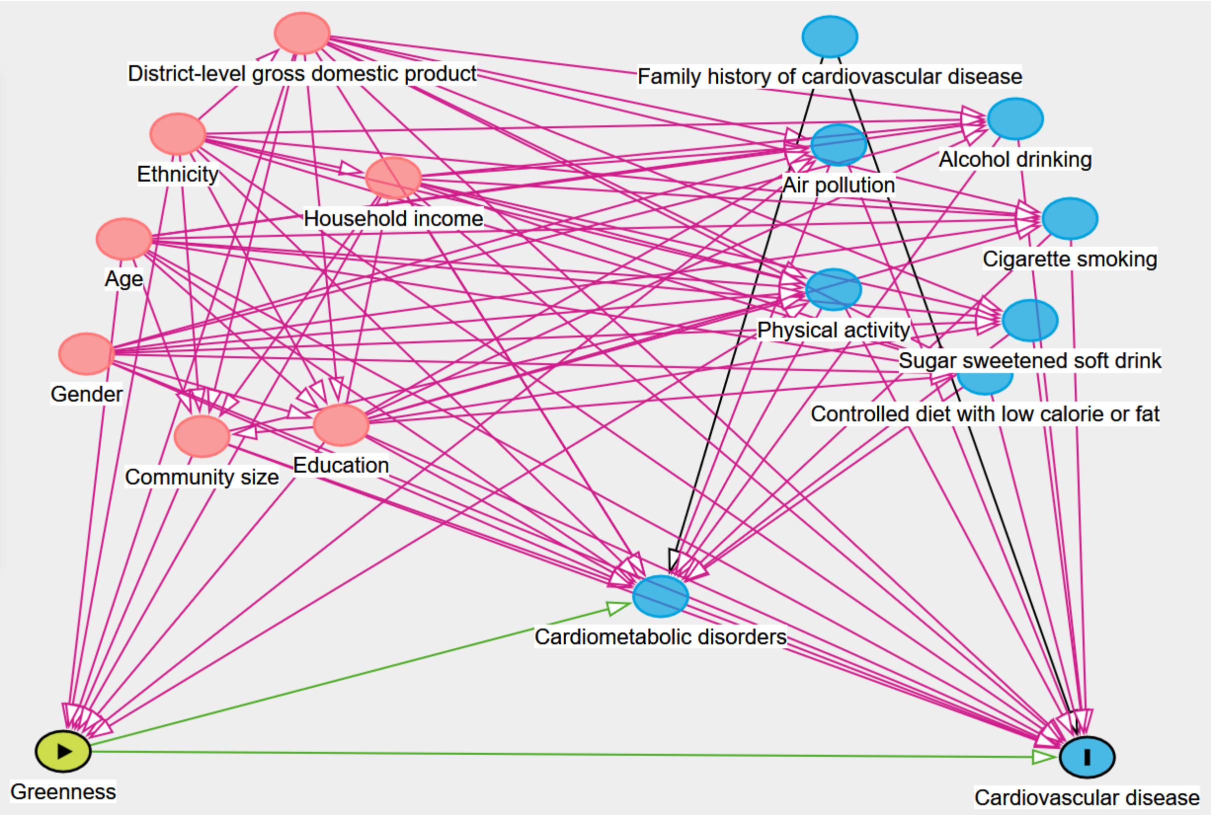

Created with the help of DAGitty.net (www.dagitty.net). Minimally sufficient adjustment set: age, sex, ethnicity, education, household income, physical activity, district-level gross domestic product, and air pollution ($PM_{2.5}$).

© 2020 Yang B-Y et al. *JAMA Network Open*.

eFigure 2. Directed Acyclic Graph for the Association Between Greenness and Cardiometabolic Disorders

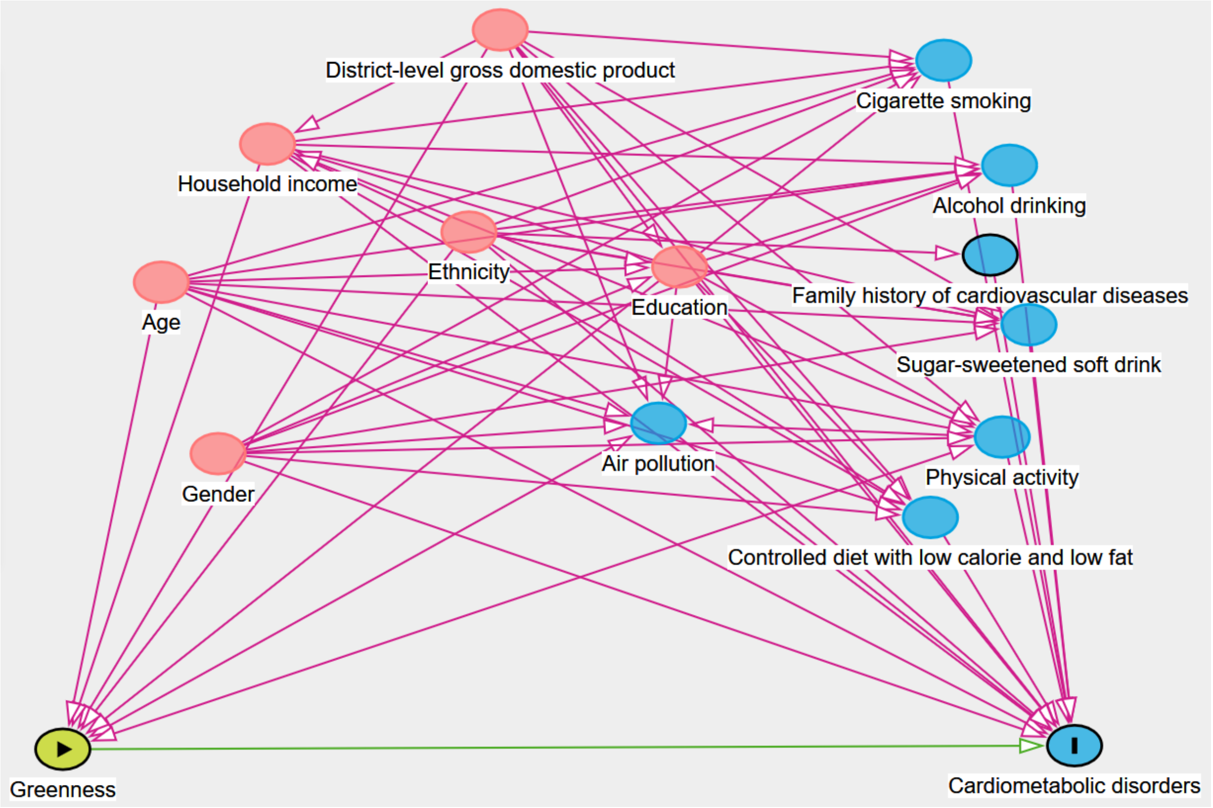

Created with the help of DAGitty.net (www.dagitty.net). Minimally sufficient adjustment set: age, sex, ethnicity, education, household income, physical activity, district-level product, and air pollution ($PM_{2.5}$).

© 2020 Yang B-Y et al. *JAMA Network Open*.

eFigure 3. Directed Acyclic Graph for the Association Between Cardiometabolic Disorders and CVD

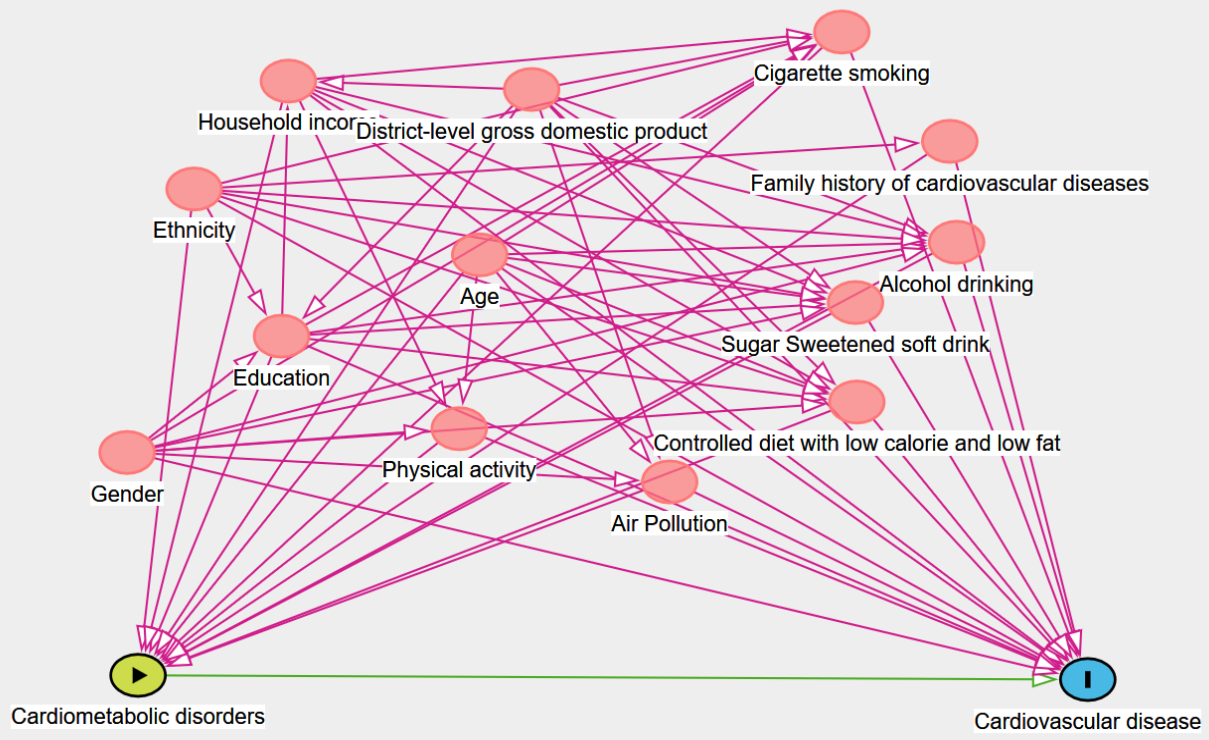

Created with the help of DAGitty.net (www.dagitty.net). Minimally sufficient adjustment set: age, sex, ethnicity, education, household income, physical activity, district-level gross domestic product, air pollution ($PM_{2.5}$), alcohol drinking, cigarette smoking, controlled diet with low calorie and low fat, sugar-sweetened soft drink, and family history of cardiovascular diseases.

© 2020 Yang B-Y et al. *JAMA Network Open*.

eFigure 4. Three-Way Decomposition Encompassing Decompositions for Both Mediation and Interaction

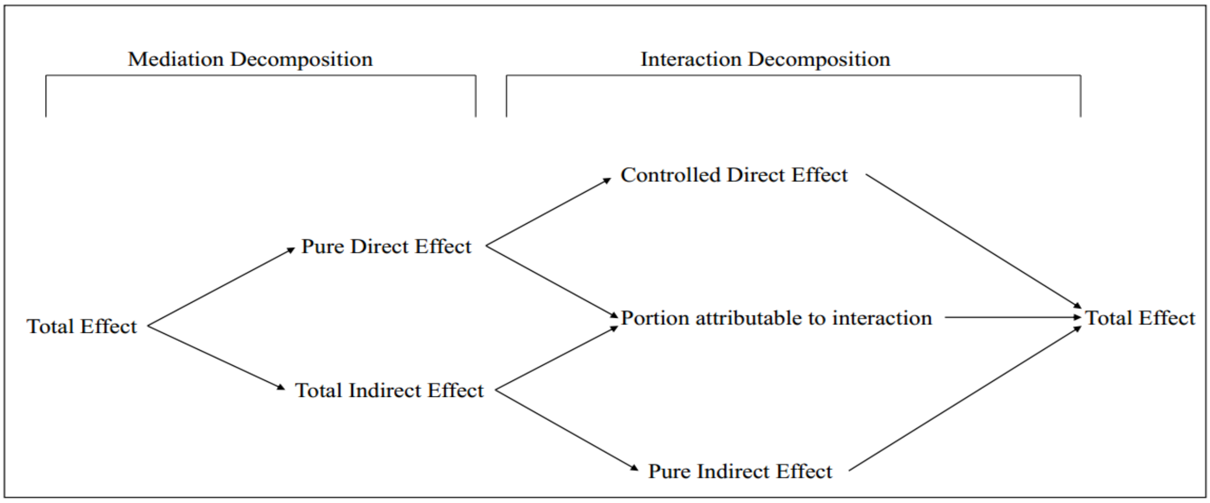

© 2020 Yang B-Y et al. *JAMA Network Open*.

eFigure 5. Dose-Response Curves for Greenness Levels With CVD Prevalence

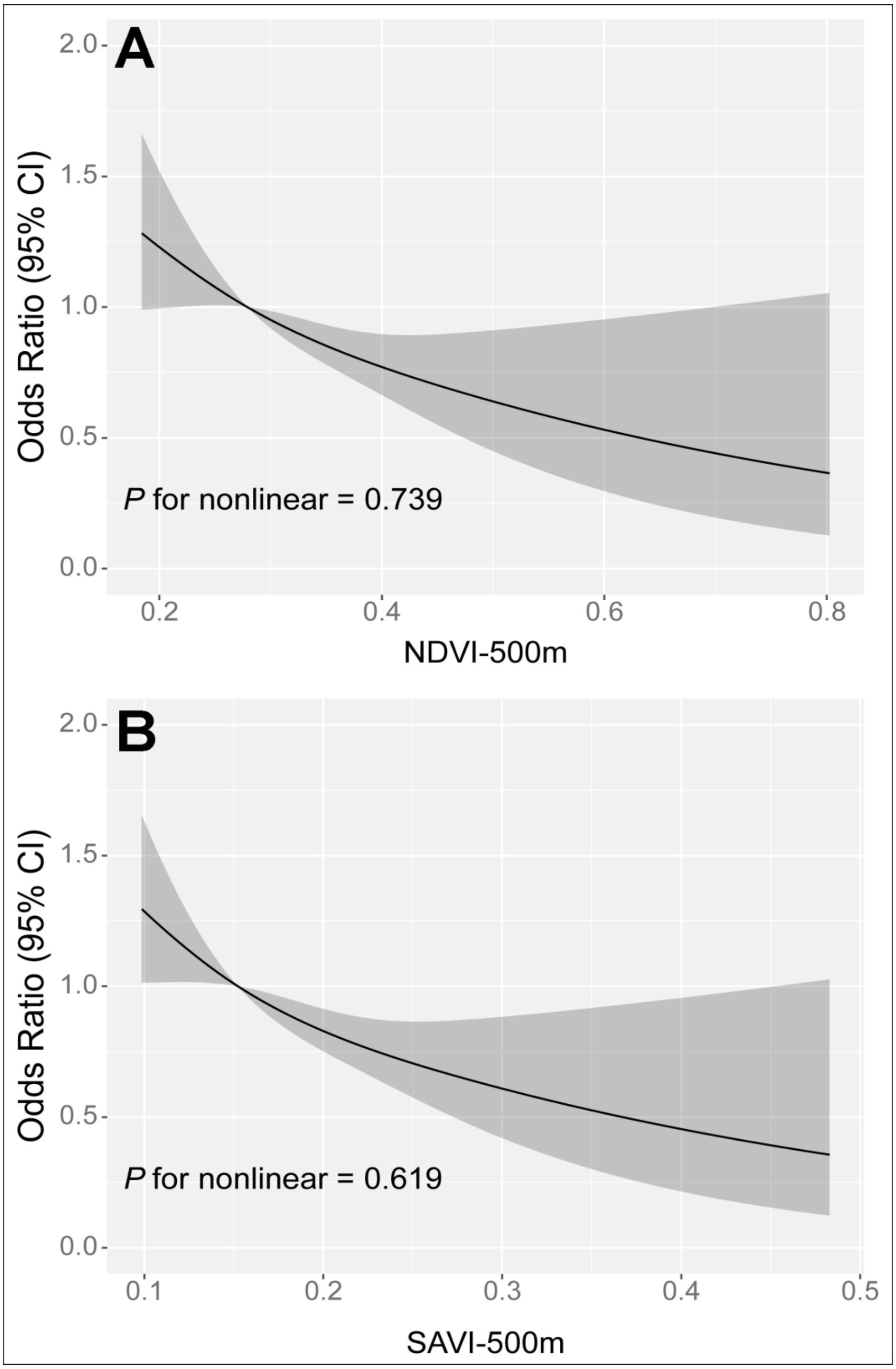

© 2020 Yang B-Y et al. *JAMA Network Open*.

eReferences

1. Hu Z, Liebens J, Rao KR (2008) Linking stroke mortality with air pollution, income, and greenness in northwest Florida: an ecological geographical study. Int J Health Geogr 7: 20.
2. Mitchell R, Popham F (2008) Effect of exposure to natural environment on health inequalities: an observational population study. Lancet 372: 1655-1660.
3. Maas J, Verheij RA, de Vries S, Spreeuwenberg P, Schellevis FG, et al. (2009) Morbidity is related to a green living environment. J Epidemiol Community Health 63: 967-973.
4. Richardson E, Pearce J, Mitchell R, Day P, Kingham S (2010) The association between green space and cause-specific mortality in urban New Zealand: an ecological analysis of green space utility. BMC Public Health 10: 240.
5. Dominguez-Berjon MF, Gandarillas A, Segura DPJ, Zorrilla B, Soto MJ, et al. (2010) Census tract socioeconomic and physical environment and cardiovascular mortality in the Region of Madrid (Spain). J Epidemiol Community Health 64: 1086-1093.
6. Coutts C, Horner M, Chapin T (2010) Using geographical information system to model the effects of green space accessibility on mortality in Florida. Geocarto International 25: 471-484.
7. Richardson EA, Mitchell R (2010) Gender differences in relationships between urban green space and health in the United Kingdom. Social Science Medicine 71: 568-575.
8. Richardson EA, Mitchell R, Hartig T, de Vries S, Astell-Burt T, et al. (2012) Green cities and health: a question of scale? J Epidemiol Community Health 66: 160-165.
9. Pereira G, Foster S, Martin K, Christian H, Boruff BJ, et al. (2012) The association between neighborhood greenness and cardiovascular disease: an observational study. BMC Public Health 12: 466.
10. Donovan GH, Butry DT, Michael YL, et al. The relationship between trees and human health: Evidence form the spread of the Emerald Ash Borer. Am J Prev Med. 2013; 44(2): 139-145.
11. Richardson EA, Pearce J, Mitchell R, Kingham S (2013) Role of physical activity in the relationship between urban green space and health. Public Health 127: 318-324.
12. Tamosiunas A, Grazuleviciene R, Luksiene D, Dedele A, Reklaitiene R, et al. (2014) Accessibility and use of urban green spaces, and cardiovascular health: findings from a Kaunas cohort study. Environ Health 13: 20.
13. Lachowycz K, Jones AP (2014) Does walking explain associations between access to greenspace and lower mortality? Soc Sci Med 107: 9-17.
14. Wilker EH, Wu CD, McNeely E, Mostofsky E, Spengler J, et al. (2014) Green space and mortality following ischemic stroke. Environ Res 133: 42-48.
15. Bixby H, Hodgson S, Fortunato L, Hansell A, Fecht D (2015) Associations between green space and health in English cities: an ecological, cross-sectional study. PLoS One 10: e119495.
16. Chum A, O'Campo P (2015) Cross-sectional associations between residential environmental exposures and cardiovascular diseases. BMC Public Health 15: 438.
17. Donovan GH, Michael YL, Gatziolis D, Prestemon JP, Whitsel EA (2015) Is tree loss associated with cardiovascular-disease risk in the Women's Health Initiative? A natural experiment. Health Place 36: 1-7.
18. James P, Hart JE, Banay RF, Laden F (2016) Exposure to Greenness and Mortality in a Nationwide Prospective Cohort Study of Women. Environ Health Perspect 124: 1344-1352.
19. Massa KH, Pabayo R, Lebrao ML, Chiavegatto FA (2016) Environmental factors and cardiovascular diseases: the association of income inequality and green spaces in elderly residents of Sao Paulo, Brazil. BMJ Open 6: e11850.
20. Ngom R, Gosselin P, Blais C, Rochette L (2016) Type and Proximity of Green Spaces Are Important for Preventing Cardiovascular Morbidity and Diabetes--A Cross-Sectional Study for Quebec, Canada. Int J Environ Res Public Health 13: 423.

© 2020 Yang B-Y et al. *JAMA Network Open*.

21. Picavet H, Milder I, Kruize H, de Vries S, Hermans T, et al. (2016) Greener living environment healthier people?: Exploring green space, physical activity and health in the Doetinchem Cohort Study. Prev Med 89: 7-14.
22. Vienneau D, de Hoogh K, Faeh D, Kaufmann M, Wunderli JM, et al. (2017) More than clean air and tranquillity: Residential green is independently associated with decreasing mortality. Environ Int 108: 176-184.
23. Wang D, Lau KK, Yu R, Wong S, Kwok T, et al. (2017) Neighbouring green space and mortality in community-dwelling elderly Hong Kong Chinese: a cohort study. BMJ Open 7: e15794.
24. Yitshak-Sade M, Kloog I, Novack V (2017) Do air pollution and neighborhood greenness exposures improve the predicted cardiovascular risk? Environ Int 107: 147-153.
25. Crouse DL, Pinault L, Balram A, Hystad P, Peters PA, et al. (2017) Urban greenness and mortality in Canada's largest cities: a national cohort study. Lancet Planet Health 1: e289-e297.
26. Jia X, Yu Y, Xia W, Masri S, Sami M, et al. (2018) Cardiovascular diseases in middle aged and older adults in China: the joint effects and mediation of different types of physical exercise and neighborhood greenness and walkability. Environ Res 167: 175-183.
27. Silveira I, Junger WL (2018) Green spaces and mortality due to cardiovascular diseases in the city of Rio de Janeiro. Rev Saude Publica 52: 49.
28. Astell-Burt T, Feng X (2019) Urban green space, tree canopy and prevention of cardiometabolic diseases: a multilevel longitudinal study of 46 786 Australians. Int J Epidemiol. Doi: 10.1093/ije/dyz239.
29. Orioli R, Antonucci C, Scortichini M, Cerza F, Marando F, et al. (2019) Exposure to Residential Greenness as a Predictor of Cause-Specific Mortality and Stroke Incidence in the Rome Longitudinal Study. Environ Health Perspect 127: 27002.
30. Kim S, Kim H, Lee JT (2019) Interactions between Ambient Air Particles and Greenness on Cause-specific Mortality in Seven Korean Metropolitan Cities, 2008-2016. Int J Environ Res Public Health 16: 1866.
31. Servadio JL, Lawal AS, Davis T, Bates J, Russell AG, et al. (2019) Demographic Inequities in Health Outcomes and Air Pollution Exposure in the Atlanta Area and its Relationship to Urban Infrastructure. J Urban Health 96: 219-234.
32. Wang K, Lombard J, Rundek T, Dong C, Gutierrez CM, et al. (2019) Relationship of Neighborhood Greenness to Heart Disease in 249 405 US Medicare Beneficiaries. J Am Heart Assoc 8: e10258.
33. Zijlema WL, Stasinska A, Blake D, Dirgawati M, Flicker L, et al. (2019) The longitudinal association between natural outdoor environments and mortality in 9218 older men from Perth, Western Australia. Environ Int 125: 430-436.

© 2020 Yang B-Y et al. *JAMA Network Open*.
